# Effect of Heat-Pressing Temperature and Holding Time on the Microstructure and Flexural Strength of Lithium Disilicate Glass-Ceramics

**DOI:** 10.1371/journal.pone.0126896

**Published:** 2015-05-18

**Authors:** Fu Wang, Zhiguo Chai, Zaixi Deng, Jing Gao, Hui Wang, Jihua Chen

**Affiliations:** 1 State Key Laboratory of Medical Stomatology, Department of Prosthodontics, School of Stomatology, Fourth Military Medical University, Xi’an, PR China; 2 State Key Laboratory of Medical Stomatology, Department of Dental Technical Laboratory, School of Stomatology, Fourth Military Medical University, Xi’an, PR China; Institute for Materials Science, GERMANY

## Abstract

The present study aimed to evaluate the influence of various heat-pressing procedures (different holding time and heat pressing temperature) on the microstructure and flexural strength of lithium disilicate glass ceramic. An experimental lithium silicate glass ceramic (ELDC) was prepared from the SiO_2_-Li_2_O-K_2_O-Al_2_O_3_-ZrO_2_-P_2_O_5_ system and heat-pressed following different procedures by varying temperature and holding time. The flexural strength was tested and microstructure was analyzed. The relationships between the microstructure, mechanical properties and heat-pressing procedures were discussed in-depth. Results verified the feasibility of the application of dental heat-pressing technique in processing the experimental lithium disilicate glass ceramic. Different heat-pressing procedures showed significant influence on microstructure and flexural strength. ELDC heat-pressed at 950℃ with holding time of 15 min achieved an almost pore-free microstructure and the highest flexural strength, which was suitable for dental restorative application.

## Introduction

Since natural teeth have inherent color and translucency [1,2], it is important that dental restorations should reproduce the optical characteristics of natural teeth. In the past several decades, the porcelain-fused- metal (PFM) restoration has been the primary selection due to the reliable mechanical properties [3,4]. However, the existence of metal, which was absolutely opaque, might lead to an esthetically unsatisfied match with natural teeth [5]. All-ceramic restorations have been advocated for super esthetic accompanied with acceptable mechanical properties [3,6–8]. Currently, several methods have been used for fabricating all-ceramic restorations [6]. Among them, heat-pressing technique was a well-established method utilizing lost-wax technique [9,10]: A wax pattern of restoration was produced and invested with refractory die material. Then the wax pattern was burnt out, and the mold cavity was filled with softened glass ceramic material which was heat-pressed at high temperature in a specific furnace under vacuum [11]. The advantages of dental heat-pressing technique compared with traditional methods for fabricating dental restorations include easy processing, increased Weibull modulus, reduced porosity and excellent marginal fitness [11–14].

Properties of ceramic materials were closely related to the microstructure and preparation technique [15,16]. Influence of dental heat-pressing treatment at certain temperature on properties of glass ceramics has been investigated previously [14,17,18]. In Albakry’s study, the heat-pressing treatment did not significantly affect the fracture toughness of Empress 1 glass ceramic, but showed influence on the fracture toughness of Empress 2 glass ceramic [17]. Repeated heat-pressing treatment could also increase the flexural strength of Empress 2 glass ceramic [18]. Luo et al revealed that, compared with traditional veneering methods, heat-pressing technique improved the transparency and lightness of zirconia-based restorations [14]. As to mica-based glass ceramic, heat-pressing treatment produced an anisotropic structure, and also improved the mechanical property in the perpendicular direction [19–21]. Results of previous studies indicated that the influence of dental heat-pressing on properties of glass ceramic was considered to be material-dependent.

Attributing to the improvement in controlled crystallization [22–24], lithium disilicate glass ceramic achieved better mechanical properties compared with other dental glass ceramics [25–29]. In the previous study, an experimental lithium disilicate glass ceramic (ELDC) in the SiO_2_-Li_2_O-K_2_O-Al_2_O_3_-ZrO_2_-P_2_O_5_ system was developed with good mechanical properties and suitable translucency for dental restorative applications [30]. As a potential restorative material, it was necessary to evaluate the ability to easily fabricate dental restorations with commercially available dental heat pressing equipment. The properties of heat-pressed mica-based glass ceramic were reported to be closely related to the heat-pressing procedures, such as temperature and holding time [20, 21]. However, no information is available about the influence of different heat-pressing procedures on properties of lithium disilicate glass ceramics in the literature. Therefore, the present study aimed to evaluate the feasibility of application of dental heat-pressing technique in processing ELDC. Moreover, the influence of various heat-pressing procedures on the microstructure and flexural strength of lithium disilicate glass ceramic was investigated, and the relationships between the microstructure, flexural strength and heat-pressing procedures were discussed in-depth.

## Materials and Methods

### Preparation of ELDC ingots

Glass in the SiO_2_-Li_2_O-K_2_O-Al_2_O_3_-ZrO_2_-P_2_O_5_ system with P_2_O_5_ content of 1.0 mol% was melted as reported previously [30]. Batches were melted in quartz crucibles at 1450°C for 2 h and the melt was quenched into distilled water to form glass frit. The as-received glass frit was milled and remelted again for better homogeneity. The melted glass was cast into preheated stainless steel moulds (at 500°C) to form cylindrical glass (12 mm in diameter and 50 mm in length), and immediately transferred to a preheated furnace for annealing at 500°C for 1 h to avoid thermal shock. Then the glass was cooled from 500°C to room temperature at a rate of 1°C min^-1^ to relieve internal stress. Subsequently, the glass rods were sectioned into small ingots (12 mm in diameter and 12 mm in height)using a low-speed cutting machine (IsoMet 1000, Buehler, USA). Following the method described in previous study, a two-stage heat treatment cycle was used for the crystallization of base glass and the glass ceramic with lithium silicate as the main crystalline phase could be formed [30].

### Heat-pressing treatment

**Fig 1:**
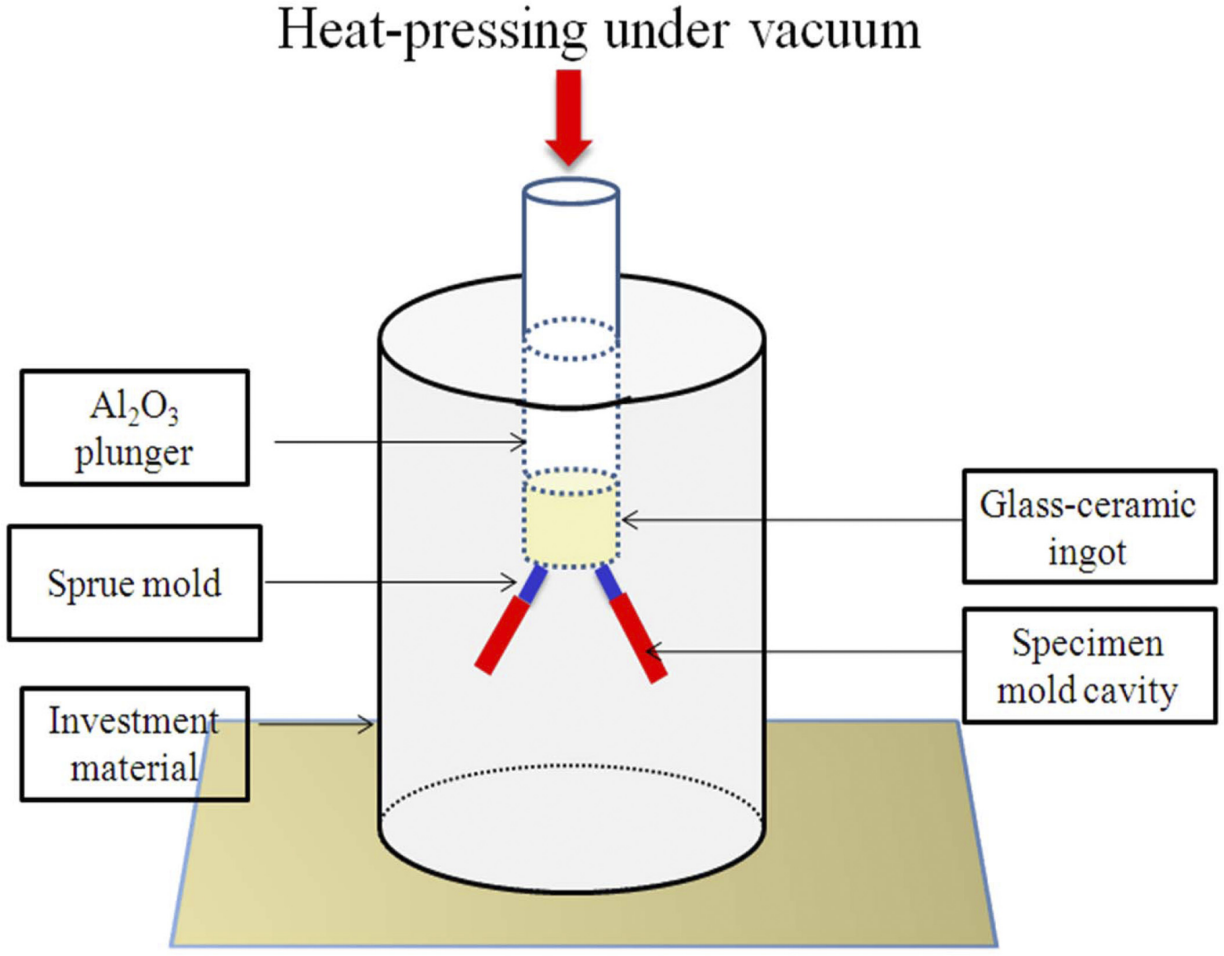
Graphical presentation of dental heat-pressing equipment.

Bar-shaped wax patterns (dimensions of 25 mm×5 mm ×2 mm) were prepared and fixed by wax sprue (3 mm in diameter). Then the wax patterns were invested with investment material (IPS PressVEST, Ivoclar Vivadent, Liechtenstein) mixed with corresponding liquid at the ratio of 100 g: 27 ml. The invested mold was transferred to a burnout furnace (Kavo EWL, Typ 5625, Germany) and heated at a rate of 5°C min^-1^ from room temperature to 850°C to melt down wax (holding for 60 min). Then the invested mold was immediately transferred into commercially available automated dental heat-pressing equipment (Dentsply Touch Press, Dentsply International, Milford, DE) which had been already preheated up to 700°C. After inserting the ELDC ingot and an alumina plunger (Fig 1), different heat pressing procedures were used by varying holding time and heat pressing temperatures (as listed in Table 1) under a pressure of 0.5 MPa. After heat-pressing treatment, the specimens were carefully devested by sand blasting with glass powders (50 μm) at a pressure of 0.2 MPa to remove investment material. Specimen surface was polished with a series of silicon carbide papers sequentially (#320, 400, 600, 800, and 1000). Length of each bar-shaped specimen was measured. The percent of material pressed defined as the length of the heat-pressed specimen divided by the length of the wax pattern (mold cavity) was calculated as descripted in the literature [31].

**Table 1:** Different heat-pressing procedures with varied temperature and holding time.

Group	Heat-pressing temperature (°C)	Holding time (min)	Pressing time (min)
**1**	940	15	5
**2**	940	5	5
**3**	950	15	5
**4**	950	5	5
**5**	960	15	5
**6**	960	5	5
**7**	970	15	5
**8**	970	5	5
**9**	980	15	5
**10**	980	5	5

### XRD and SEM

The crystalline phases of the glass ceramics were characterized by X-ray diffraction (XRD). Samples were ground to fine powders and then analyzed using X-ray diffractometer (D/Max2550VB, Rigaku International Corp, Japan) with Cu Kα radiation. Voltage and current were selected as 40 kV and 20 mA, respectively. Data were collected from 2θ = 10° to 70° at a scanning rate of 1° min^-1^. Highly polished surface of glass ceramic specimens were etched with 5% hydrofluoric acid solution for 60 s. After ultrasonic cleaning, specimens were gold sputtered and the microstructure was observed using scanning electron microscopy (SEM) (JSM-7000F, JEOL, USA).

### Flexural strength measurement

Following the instructions of ISO 6872 [32] established for dental ceramics, specimens were ground and polished with a series of silicon carbide papers sequencely (#320, 400, 600, 800 and 1000) to produce bar-shaped specimens with dimensions of 25 mm×4 mm×1.2 mm, which was checked with a digital micrometer with an accuracy of ±0.01mm. Before measurement, specimens were ultrasonically cleaned in distilled water for 10 min and dried with compressed air. Three-point flexural strength was measured with a universal testing apparatus (AGS-10kNG, Shimadzu, Japan), at a span length of 15 mm and a cross-head speed of 0.5 mm min^-1^. The flexural strength (M) was calculated from the equation listed below:

$$M=\frac{3Wl}{2bd^{2}}$$

where *W* is the breaking load, *l* is the test span, *b* is the width and *d* is the thickness of the specimen. Data were analyzed using one-way analysis of variance (ANOVA) followed by Turkey HSD test using statistic software (SPSS 12.0, SAS, Chicago, Ill, USA; α = 0.05)

## Results and Discussion

As a candidate restorative material for dental application, ELDC should achieve the challenging requirements in restorative dentistry: ease of fabrication and adequate mechanical property [9]. Heat-pressing technique used in the present study has been considered as easy processing, less time-consuming and with excellent marginal fitness [11–14]. Viscosity of glass ceramic at certain temperature was crucial to the ability to be heat-pressed. However viscosity was very difficult to be measured especially at high temperature. Therefore percent of material pressed, defined as the length of the produced specimen divided by the length of the wax pattern (mold cavity) [31], was measured in the present study to evaluate the feasibility of the experiment material for fabricating dental restoratives using heat-pressing technique. Results demonstrated that almost all groups produced excellent pressed percent, except for 940°C groups in which the mold cavity was not completely filled (Table 2). This indicated that the viscosity of experimental glass ceramic at 940°C was not low enough to complete the filling procedure during the heat-pressing time. Based on results of the present study, the feasibility of application of dental heat-pressing technique in processing ELDC was verified. In Gorman CM’s study, longer holding time led to greater filling content when a fluorapatite glass was heat-pressed [21]. Prolonged holding time was also proved to be useful for developing anisotropic structure and improving mechanical properties of a mica-based glass ceramic [20]. In the present study, similar results were also observed in the lithium disilicate glass ceramic. Although insufficient filling was observed 940°C groups, longer holding time (15 min) achieved a greater pressed percent (84%). For specimens heat-pressed at 950°C and 960°C, prolonging holding time improved the flexural strength significantly (p<0.05) (Table 2).

**Table 2:** The percent of material pressed and flexural strength after different heat-pressing procedures.

Group	Heat-pressing temperature (°C)	Holding time (min)	Percent of pressed (%)	Flexural strength (MPa)*
**1**	940	15	84	NA
**2**	940	5	71	NA
**3**	950	15	100	285±11^a^
**4**	950	5	100	245±21^b^
**5**	960	15	100	248±17^b^
**6**	960	5	100	220±21^c^
**7**	970	15	100	210±17^c^
**8**	970	5	100	220±16^c^
**9**	980	15	100	179±29^d^
**10**	980	5	100	184±24^d^

*Same letters indicated no statistical difference (p>0.05); NA: not available.

**Fig 2:**
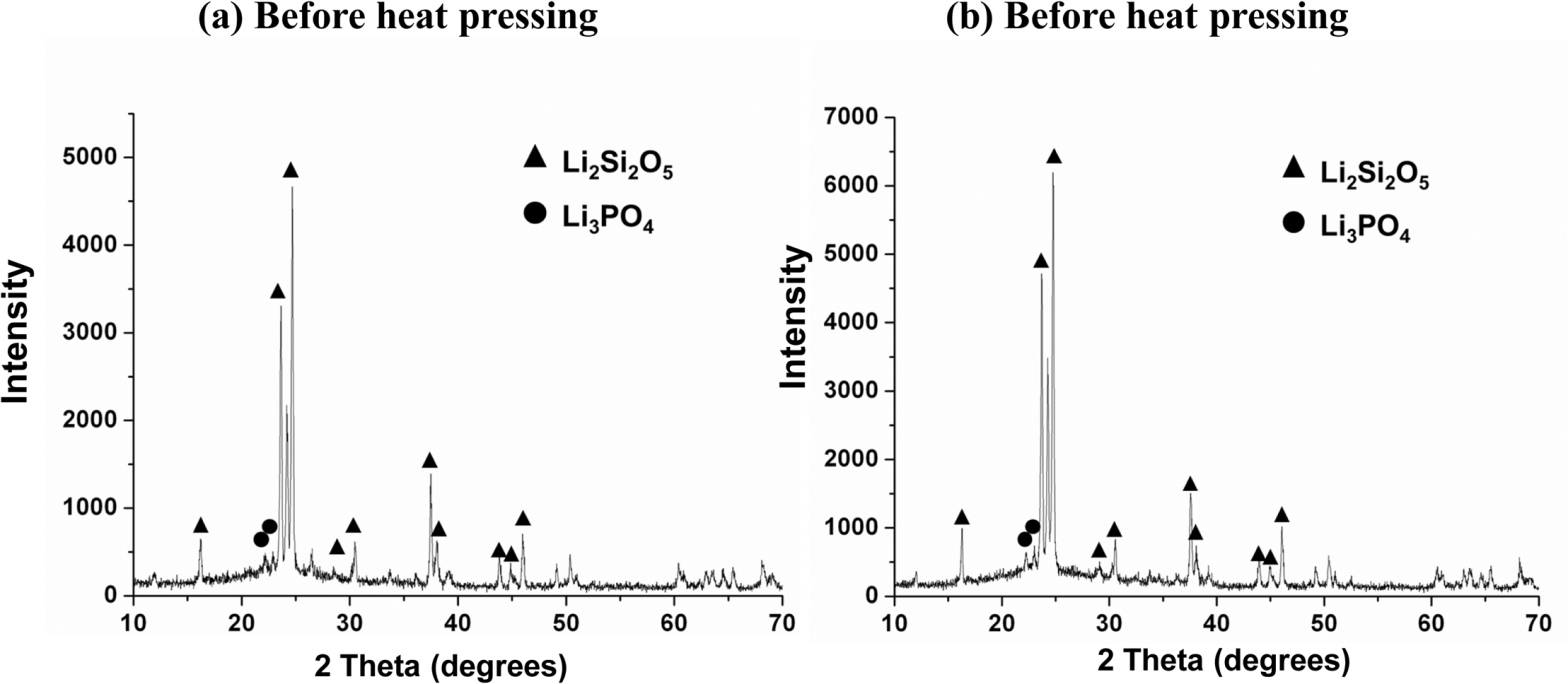
X-ray diffraction patterns of experimental lithium disilicate glass ceramic. (a) before heat-pressing. (b) after heat-pressing at 950°C. No change in crystalline compositions was observed. Lithium disilicate (Li_2_Si_2_O_5_) was represented as the major crystalline phase. The intensity of the main diffraction peak increased after heat-pressing (b).

**Fig 3:**
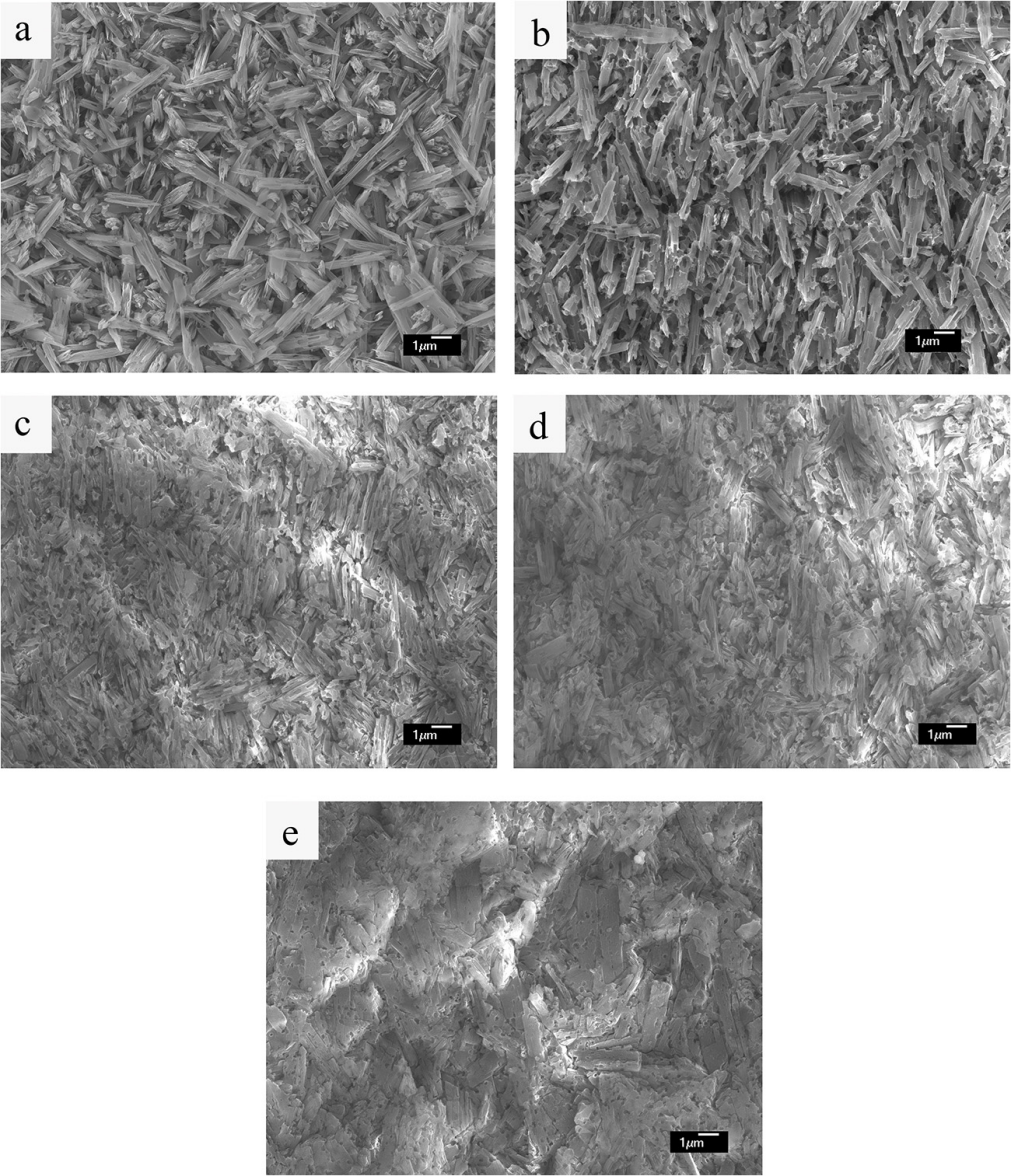
SEM photographs of the etched polished surface of lithium disilicate glass ceramics. (a) before heat-pressing, (b) heat-pressed at 950°C, (c) heat-pressed at 960°C, (d) heat-pressed at 970°C, (e) heat-pressed at 980°C. Crystal size of ELDC showed a small extent of growth as heat-pressing temperature increased and crystal alignment along the direction of pressing was observed in the heat-pressed specimens.

Mechanical properties of dental glass ceramics related closely to crystalline composition and microstructure [6]. In the present study, XRD results found no significant change in the crystalline composition for ELDC before and after heat-pressing treatment (Fig 2). Lithium disilicate (Li_2_Si_2_O_5_) was represented as the major crystalline phase, together with a small amount of lithium phosphate (Li_3_PO_4_), which was considered to be the nucleation site during crystallization of lithium disilicate glass [23,24,33,34]. Results of the present study also confirmed Gorman CM’s research in which full crystallization of lithium disilicate glass ceramic system was already achieved before heat-pressing treatment [13]. The microstructure of ELDC before and after heat-pressing treatment at different temperature was demonstrated in Fig 3. Specimens before heat-pressing exhibited randomly oriented lithium disilicate crystals within the glassy matrix (Fig 3A). While in heat-pressed specimens, a tendency of crystal alignment along the direction of pressing was observed and the crystal size showed a small extent of growth as heat-pressing temperature increased (Fig 3B–3E). This anisometric microstructure might be as the result of the shearing force inside the softened material when it flowed through the narrow sprues during heat-pressing procedure [9,35]. The origination of crystals along the direction of heat-pressing would make cracks propagating in the perpendicular direction following a more tortuous path, thereby improving fracture toughness [20]. Another fact was that crystals were not only aligned along the direction of pressing, but also parallel to the surface of the pressed specimens. As the case of heat-pressed dental restorations, the crystal alignment was most likely to be parallel to the contour curve of glass ceramic restorations. Therefore an increased resistance to the vertical force might be expected from such kind of structure.

**Fig 4:**
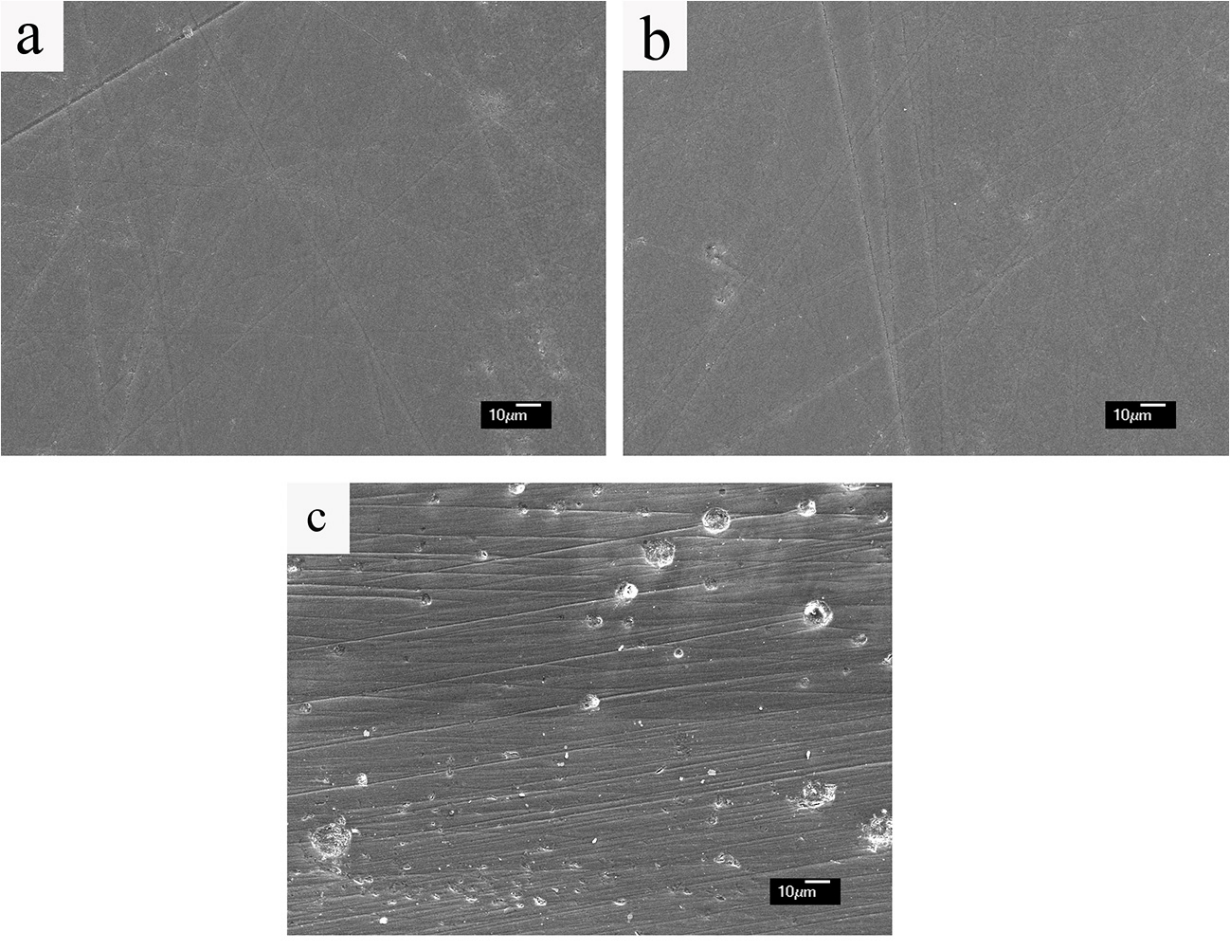
SEM images of polished surface of experimental glass ceramics. (a) heat-pressed at 950°C, (b) heat-pressed at 960°C, showing almost no pores; (c) heat-pressed at 980°C, showing distribution of some spherical pores.

Presence of pores in the bulk or surface of dental ceramic had a detrimental influence on the flexural strength [36]. Therefore porosity control should be a fundamental consideration during fabricating dental restoratives using glass ceramic, in order to reduce frequency of fracture of dental ceramic restorations when chewing hard food. Specimens heat-pressed at 950°C and 960°C was found to be almost free of pores in the surface (Fig 4A and 4B). This pore-free microstructure was mainly attributed to the densification effect of softened glass ceramic pressed at high temperature under vacuum [13]. As for specimens heat-pressed at 980°C, obvious pores were found (Fig 4C). A possible explanation was that increased fluidity of softened glass ceramic at 980°C might lead to non-pressed filling during holding time. As a consequence of the loss of pressing effect, air bubble might involve into the specimens. Results of the present study identified significant influence of heat-pressing temperature and holding time on the flexural strength of lithium disilicate glass ceramic (Table 2). This was in well agreement with Zhao’s study in which heat-pressing temperature showed influence on the mechanical properties of mica-based glass ceramic [20]. As the temperature increased, the flexural strength decreased lightly. A possible explanation was the growth of crystalline size. Porosity played a detrimental effect on the flexural strength by acting as a stress concentrator [36]. This would be one of the factors contributed to the lowest flexural strength of specimens heat-pressed at 980°C.

Based on the results of present study, the feasibility of application of dental heat-pressing technique in processing the experimental lithium disilicate glass ceramic was verified. The flexural strength of heat-pressed ELDC fully met the requirements established for dental restorative ceramics in ISO 6872 [32] and was also comparable with that of commercial dental glass ceramics reported in the literature [37,38]. Moreover, it was indicated that besides the inherent characteristics of glass ceramics, the final outcome of ceramic restorations was also sensitive to the heat-pressing procedures utilized. Any minute change of heat-pressing temperature might lead to different outcome properties, which should be taken into consideration when pressable glass ceramics were used in clinical dentistry. The clinical performance of glass ceramic restorations was also influenced by other factors, such as the ability to bond with teeth, different surface treatments and chemical resistance in oral environment. Further research need to be carried out considering the above mentioned factors.

## Conclusions

With the limitations of the present study, the outcomes can be summarized as follows:

1. The feasibility of application of dental heat-pressing technique in processing ELDC was verified. An anisotropic and pore-free microstructure could be obtained by commercially available dental heat-pressing equipments.
2. Dental heat-pressing treatment did not change the crystalline composition of lithium disilicate glass ceramic, but crystals demonstrated some kind of orientation along the pressing direction.
3. Different heat-pressing temperature and holding time had significant influence on microstructure and flexural strength of the lithium disilicate glass ceramic. With the limitations of present study, ELDC heat-pressed at 950°C with a holding time of 15 min achieved almost pore-free microstructure and the highest flexural strength.
