# Correction: Effect of Heat-Pressing Temperature and Holding Time on the Microstructure and Flexural Strength of Lithium Disilicate Glass-Ceramics

**DOI:** 10.1371/journal.pone.0130919

**Published:** 2015-06-12

**Authors:** Fu Wang, Zhiguo Chai, Zaixi Deng, Jing Gao, Hui Wang, Jihua Chen

There are errors in affiliation 1 for authors Fu Wang, Zhiguo Chai, Jung Gao, Hui Wang, and Jihua Chen and affiliation 2 for author Zaixi Deng.

Affiliation 1 should be: State Key Laboratory of Military Stomatology, Department of Prosthodontics, School of Stomatology, Fourth Military Medical University, Xi’an, PR China

Affiliation 2 should be: State Key Laboratory of Military Stomatology, Department of Dental Technical Laboratory, School of Stomatology, Fourth Military Medical University, Xi’an, PR China

## References

[1] Wang F, Chai Z, Deng Z, Gao J, Wang H, Chen J (2015) Effect of Heat-Pressing Temperature and Holding Time on the Microstructure and Flexural Strength of Lithium Disilicate Glass-Ceramics. PLoS ONE 10(5): e0126896 doi:10.1371/journal.pone.0126896 25985206 10.1371/journal.pone.0126896 PMC4436011

